# Evaluation of deceased-donor kidney offers: development and validation of novel data driven and expert based prediction models for early transplant outcomes

**DOI:** 10.3389/fimmu.2024.1511368

**Published:** 2025-01-07

**Authors:** Christoph F. Mahler, Felix Friedl, Christian Nusshag, Claudius Speer, Louise Benning, Daniel Göth, Matthias Schaier, Claudia Sommerer, Markus Mieth, Arianeb Mehrabi, Christoph Michalski, Lutz Renders, Quirin Bachmann, Uwe Heemann, Markus Krautter, Vedat Schwenger, Fabian Echterdiek, Martin Zeier, Christian Morath, Florian Kälble

**Affiliations:** ^1^ Department of Nephrology, University Hospital Heidelberg, Heidelberg, Germany; ^2^ Department of General, Visceral and Transplantation Surgery, University Hospital Heidelberg, Heidelberg, Germany; ^3^ Department of Nephrology, Klinikum Rechts der Isar, Technische Universität München (TUM), Munich, Germany; ^4^ Department of Nephrology, Hospital Stuttgart, Stuttgart, Germany

**Keywords:** kidney transplantation, donor selection criteria, graft loss, donor score, kidney donor risk index (KDRI)

## Abstract

In the face of growing transplant waitlists and aging donors, sound pre-transplant evaluation of organ offers is paramount. However, many transplant centres lack clear criteria on organ acceptance. Often, previous scores for donor characterisation have not been validated for the Eurotransplant population and are not established to support graft acceptance decisions. Here, we investigated 1353 kidney transplantations at three different German centres to develop and validate novel statistical models for the prediction of early adverse graft outcome (EAO), defined as graft loss or CKD ≥4 within three months. The predictive models use generalised estimating equations (GEE) accounting for potential correlations between paired grafts from the same donor. Discriminative accuracy and calibration were determined via internal and external validation in the development (935 recipients, 309 events) and validation cohort (418 recipients, 162 events) respectively. The expert model is based on predictor ratings by senior transplant nephrologists, while for the data-driven model variables were selected via high-dimensional lasso generalised estimating equations (LassoGee). Both models show moderate discrimination for EAO (C-statistic expert model: 0,699, data-driven model 0,698) with good calibration. In summary, we developed novel statistical models that represent current clinical consensus and are tailored to the older deceased donor population. Compared to KDRI, our described models are sparse with only four and three predictors respectively and account for paired grafts from the same donor, while maintaining a discriminative accuracy equal or better than the established KDRI-score.

## Introduction

Kidney transplantation is the gold-standard for patients with end-stage renal disease (1, 2). In Germany 1517 cadaveric kidney transplantations were performed in 2021, while 6593 patients remain listed for kidney transplantation (3). In view of this pressing donor shortage optimal organ acceptance strategies are essential (4).

However, despite numerous studies, particularly in western countries, the role of donor characteristics and how to best integrate them into graft acceptance strategies remains largely unknown (5). Yet, donor cues associated with an increased risk of early graft failure have been established including age, comorbidity, immunologic and genetic factors (6–8). Conversely, a registry-based study investigated the role of donor-associated risk in donor-kidney pairs and revealed that once both kidneys are considered eligible for transplantation, donor factors have minimal effect on early transplant outcome (9, 10). This indicates clinical decisions to accept or decline an organ offer are largely efficient.

Hence investigating which donor factors educate the clinical decision process and how they impact early transplant outcome is vital to understanding the role of donor characteristics and the development of standardised acceptance criteria.

Here we conducted an in-depth analysis of the deceased-donor characteristics of 1353 kidney transplantations at three German Eurotransplant centres concerning their impact on early adverse outcome (EAO) which we defined as early graft loss or impaired kidney function (CKD ≥ 4; eGFR < 30 ml/min) three months after transplantation. This approach thus eliminated the majority of bias attributed to variable and long-term environmental influences like post-transplant care and recipient behaviour (9, 11). The rationale for EOA as primarily donor- and procedure driven is echoed by transplant policies allowing recipients immediate reinstatement of accrued waiting time after graft failure within 90 days after transplantation (12).

For a baseline and comparison, we first calculated KDRI scores and performed model calibration for prediction EOA. Subsequently, we developed different new models based on previously suggested variables, *data-driven variable selection* and *expert input* from senior nephrologists. These new models were internally and externally validated for prediction of EOA within three months from transplantation.

## Materials and methods

### Study cohort

In this study we retrospectively included 1353 deceased kidney donor transplantations between 2006 and 2021 at our centre (605 grafts), Stuttgart transplant centre (418 grafts) as well as the transplant centre of the Technical University in Munich (330 grafts). Partner grafts were defined as transplantations where both kidneys from a single donor were transplanted in different individuals, this paired nature of the data was accounted for in the statistical models (10). We collected donor and recipient characteristics with their respective clinical outcome after transplantation. The local Ethics Committees authorised the study without a requirement for individual consent. The following inclusion criteria were applied: Recipients aged 18 years or older, offer of a kidney-organ from a deceased donor via Eurotransplant, transplantation of both kidneys from a single donor at the same centre. Exclusion criteria were combined organ offers (heart-kidney, pancreas-kidney).

### Outcome

We defined early adverse outcome (EAO) as a composite of graft loss or CKD ≥4 within three months after transplantation. We reasoned that donor related graft function might be most prominent during the early phase post transplantation, whereas recipient and environmental factors mainly bias late graft failure. The CKD-EPI formula without race (13) was used for calculation of donor eGFR and recipient eGFR after transplantation.

### Statistical analysis

The data collection in the context of the presented project was performed with the help of an electronic database system (Microsoft Excel 2018, Microsoft Germany GmbH, Unterschleißheim). A statistical evaluation was then carried out using RStudio (R team 2021).

There was no missing data for three month graft function, graft survival or three month recipient survival. There was less than 1,5% of overall data missing. Merged multiple imputation was used to compute the mean of all imputed values of each missing value.

### Variable selection and clustered analysis

Univariate Generalised Estimating Equation (GEE) (14, 15) models were employed to calculate odds ratios (OR) and confidence intervals (CI) for individual variables (16). For variable selection we applied high-dimensional lasso generalised estimating equations (LassoGee), to identify significant predictors (17). 1000-fold bootstrapping was used to ensure the stability and reliability of the selected variables. A multivariate GEE model was then fitted based on the previously selected variables. The Kidney Donor Risk Index (KDRI) was calculated using standard clinical parameters (18–20) and a cox model was re-calibrated. For internal validation we used 250-fold bootstrapping analysis, as recommended by the transparent reporting of a multivariable prediction model for individual prognosis or diagnosis guidelines.

## Results

### Baseline characteristics and study cohort

1353 kidney transplantations at three different Eurotransplant centres from 1184 donors were included for analysis, 605 at centre 1 (Heidelberg), 330 in centre 2 (Munich) and 418 at centre 3 (Stuttgart) between the years 2006-2021. Donor and recipient characteristics are summarised in **Table 1**. More recipients were male with a median of age of 62 and a median of BMI of 25. Few of them were sensitised (median/IQR of vPRA > 5% 0/5). Most patients received tacrolimus (70%) as initial immunosuppression. Patients on cyclosporine A are patients at the beginning of the study period, where cyclosporine A was more commonly used, especially within the ESP program. Within the donors, male and female was equally distributed, median age was 60 with a median BMI of 27.

**Table 1:** Donor and recipient characteristics.

Kidney transplant recipients (N=1353)		
Gender (male)	887 (66)
Age	62 (16)
Diabetes	226 (18)
Hypertension	1012 (82)
BMI^a^	25 (6)
Time on dialysis (years)	6 (6)
vPRA^b^ >5%	0 (5)
HLA^c^ Mismatches	
0	108 (8)
1-4	914 (68)
>4	327 (24)
Immunosuppressive medication	
Tacrolimus	917 (69)
Ciclosporin A	413 (31)
Delayed graft function	434 (32)
Cold Ischemia Time (hours)	12 (8)

Donors (N=1184)*		
Gender (male)	587 (50)
Age	60 (21)
Diabetes	147 (12)
Hypertension	606 (51)
BMI	27 (5)
Smoker	473 (40)
Minimal eGFR^d^ (ml/min)	92 (28)
Creatinine at explantation	1.05 (0.5)
Duration of Resuscitation (min)	18 (15)
Cause of brain death prior donation	
Stroke	758 (64)
Others	426 (36)

Demographic data from all 1353 kidney transplant recipients and 1184 donors.

(*169 donor kidney pairs).

Data is given in median (IQR) or number (percent). a: body-mass-index, b: virtual Panel-Reactive Antibody, c: Human Leukocyte Antigen, d: estimated glomerular filtration rate (CKD-Epi-formula).

For some variables data are missing, therefore N’s may not sum up to the total number of recipients/donors included.

**Figure 1:**
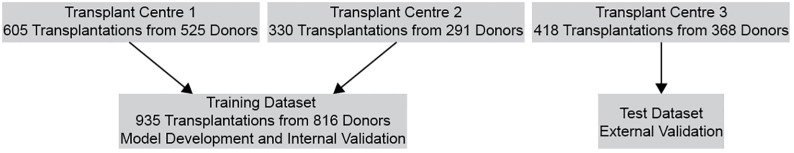
Flow chart. Flow diagram of the statistical evaluation. Deceased donor kidney transplantations at three centres between 2006 and 2021 were collected (grey boxes). Two centres were combined for model development and internal validation, the third centre was used as an external validation dataset.

The data was split into separate cohorts, where 935 performed transplantations from centre 1 and 2 were used for model development and internal validation. The transplant outcomes at centre 3 were used for external validation, see **Figure 1**. In both cohorts, 9% (n= 83 and n=36 respectively) of the recipients experienced death censored graft failure, while impaired graft function (eGFR <30) was more common in the validation cohort (30%, n= 126) than in the development cohort (24%, n=226) at three months.

### Model development

To assess whether we can identify donor factors with a substantial impact on early transplant function we used EAO as a composite outcome of graft failure and CKD ≥4. Potential donor associated predictors were selected by the authors based on the existing literature, data availability and suggestions by senior nephrologists. All donor variables were included in a *full statistical model* (see **Supplementary Table 1**) for the prediction of EAO (apart from height and weight as these correlate directly with the BMI). Through Lasso GEE the coefficients for donor age, donor eGFR and cause of brain death were estimated as non-zero and therefore included in the *data-driven model* (see **Supplementary Table 2**).

**Figure 2:**
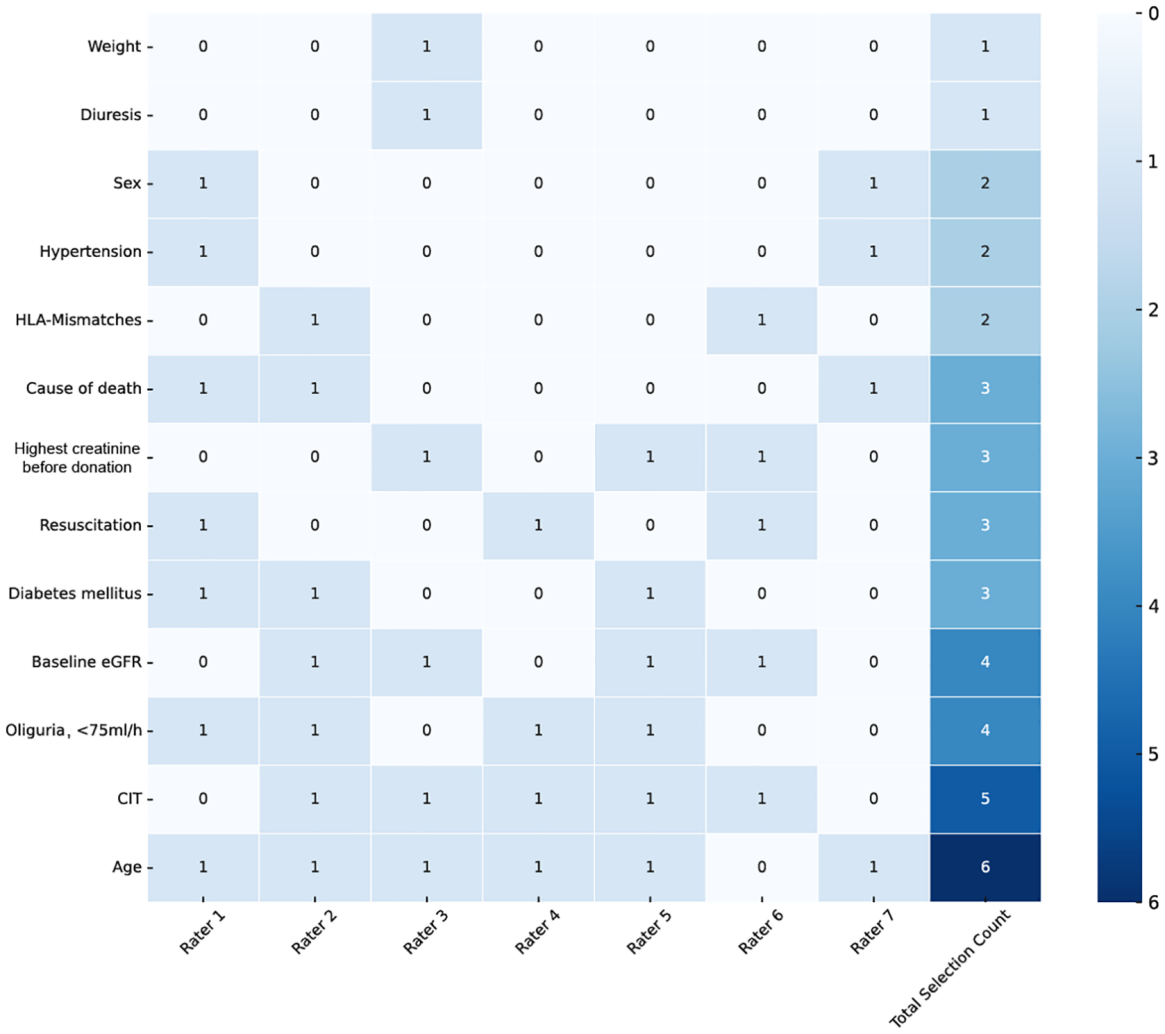
Expert selection. Clinical expert based variable nomination. Nominations of donor characteristics with clinical relevance for graft acceptance and presumed impact on early transplant function. Raters one to seven represent individual senior transplant nephrologists from all three centres. Variables with at four nominations were selected for further analysis. HLA, human leucocyte antigen; eGFR, estimated glomerular filtration rate; CIT, cold ischemic time.

An *expert model* (see **Supplementary Table 3**) was developed based on a survey among seven senior transplant nephrologists from all participating centres. They were asked to select the most valuable donor factors in predicting 90-days kidney function. Four potential donor factors (age, minimal eGFR before donation, urine output and cold ischemic time (CIT) were preselected based on clinical expert majority opinion (>50%) **Figure 2**. All models are summarised in **Table 2**.

**Table 2:** Comparison of models and variables used.

Number of variables included	Full model	Data-driven model	Expert model	KDRI model*
	16	3	4	7
Donor Variable				
Age	✓	✓	✓	✓
Minimal eGFR	✓	✓	✓	
Creatinine at explantation	✓			✓
Cause of brain death	✓	✓		✓
Urine output	✓		✓	
Body-Mass-Index	✓			
Hypertension	✓			✓
Diabetes	✓			✓
Resuscitation	✓			
Height				✓
Weight				✓
Smoker	✓			
Ongoing renal replacement therapy	✓			
Length of stay	✓			
Cold ischemia time	✓		✓	
HLA mismatch	✓			

*Race, HCV status and DCD (Donation after cardiac death) status were “hardcoded” as “no” for KDRI calculation and neither were present in our donor cohort. Height and weight excluded from full model due to correlation with BMI. Full model includes all variables selected by the research team as described above apart from height and weight. The variables in the data-driven model were selected based on LassoGee, whereas the variables in the expert model were included based on the expert ratings. The variables in KDRI model were based on the publicly available coefficients for the KDRI score.

### Discriminative ability of different statistical models

**Figure 3:**
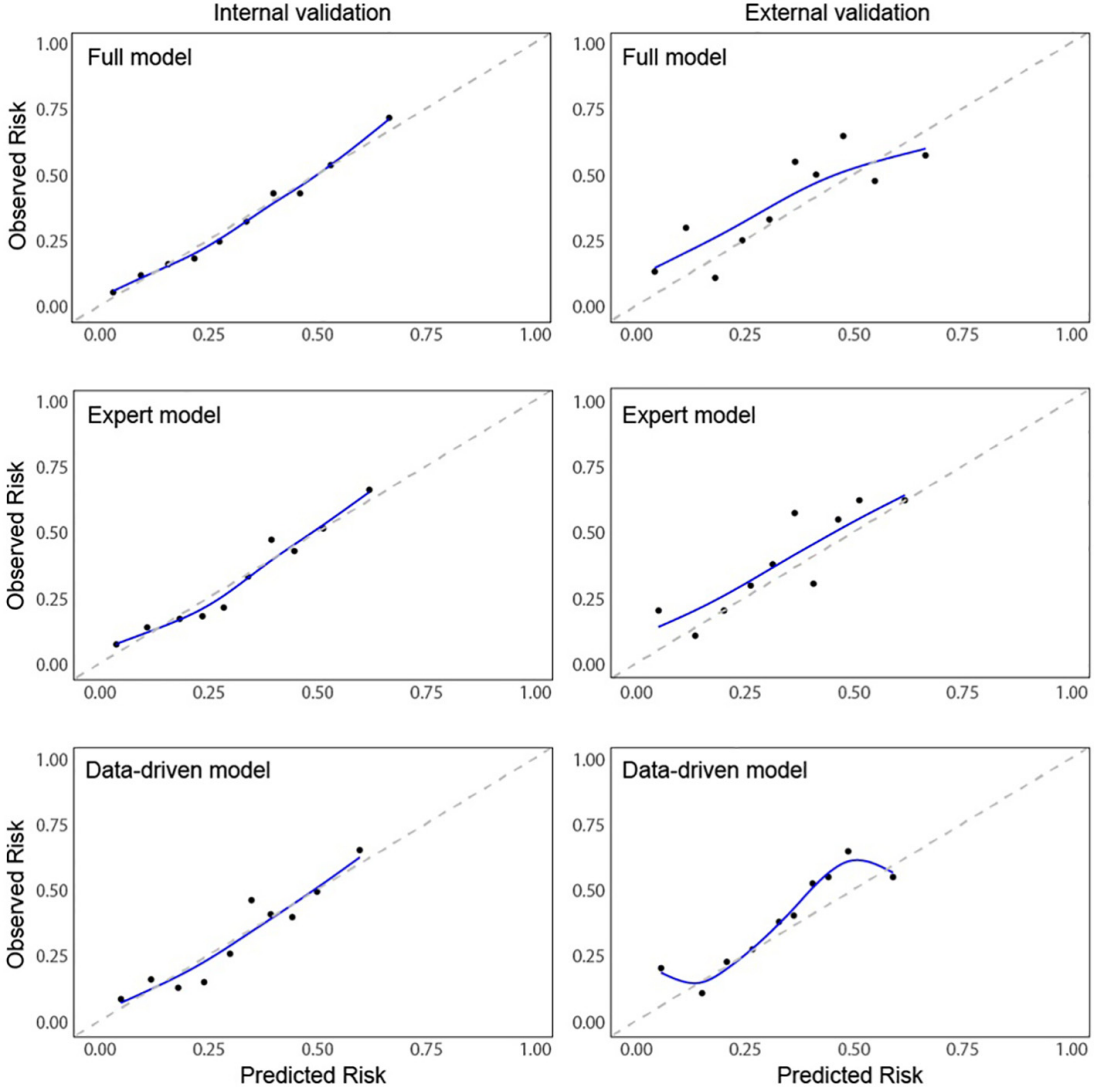
Calibration plots of early adverse outcome (EAO). Calibration Curves of Early Adverse Outcomes (EAO) for Internal and External Validation. This figure displays calibration plots comparing predicted versus observed risks for kidney transplant failure across three different models (Full, Expert and Data-driven) using both internal and external validation data. The left column shows calibration curves for internal validation, while the right column shows calibration curves for external validation. Each plot presents decile-based calibration points: Mean predicted risk (x-axis) and mean observed risk (y-axis) within each decile. Smoothed line (blue): A Generalized Additive Model (GAM) fit, providing a smooth approximation of the calibration relationship. Dashed diagonal line (gray): Represents perfect calibration, where predicted and observed risks are equal. Each curve illustrates the agreement between predicted and observed outcomes, with better calibration reflected by points and the blue line aligning closely with the diagonal reference line.

All assessed models showed moderate predictive accuracy. The C-statistic of the full-model was 0.727 and 0.689 for the internal and external validation respectively, which is similar to the cox model based on KDRI scores (0.713 and 0.687). The *data-driven model* showed improved C-statistics of 0.728 and 0.698 respectively and had almost identical C-statistics as the *expert model* of 0.718 and 0.699, respectively. In both, the *data-driven* and the *expert model* calibration was generally good and improved compared to the full model, although the models showed a tendency to underpredict risk, particularly for high-risk patients (**Table 3**; **Figure 3**).

**Table 3:** Comparison of models in external and internal validation.

Model performance	Development	Internal validation	External validation
Full model			
C-statistics (95% CI)	0.795 (0.764-0.825)	0.727^#^	0.686 (0.635-0.738)
Calibration slope	1	1.001	0.654
Calibration intercept	0	0.001	0.252
Calibration in the large, %*	33.0 vs. 33.0	33.64 vs 33.00	34.5 vs. 38.75
Data-driven model			
C-statistics (95% CI)	0.725 (0.665-0.815)	0.728^#^	0.698 (0.647-0.749)
Calibration slope	1	0.999	0.789
Calibration intercept	0	<0.001	0.258
Calibration in the large, %*	33.0 vs. 33.0	33.03 vs 33.00	34.24 vs. 38.75
Expert model			
C-statistics (95% CI)	0.720 (0.636-0.792)	0.718^#^	0.699 (0.648-0.750)
calibration slope	1	0.999	0.824
calibration intercept	0	<0.001	0.268
calibration in the large, %*	33.0 vs. 33.0	33.04 vs 33.00	33.94 vs. 38.75
KDRI model			
C-statistics (95% CI)	0.711 (0.634-0.786)	0.713^#^	0.687 (0.635-0.739)

C-statistics (95% CI): The area under the receiver operating characteristic curve (AUC), assessing the model’s ability to discriminate between outcomes. Calibration slope: Reflects how well predicted probabilities align with actual outcomes. A slope of 1 indicates perfect calibration. Calibration intercept: The difference between predicted and observed probabilities. Calibration in the large, %: Shows the overall agreement between predicted and observed probabilities, presented as percentages. ^#^No CI 95% calculated as based on bootstrap analysis, *Calibration in the large is given as predicted vs. observed. CI, confidence interval.

Analysis of Variance (ANOVA) statistics for model comparison revealed that the *data-driven model* provides the best balance between prediction capability and complexity with significant improvements over the KDRI model which performed worst in our cohort. Correspondingly the Bayesian Information Criteria (BIC) for KDRI was significantly increased compared to the data-driven model (1146.8, 1072.8 respectively). The statistical model with all parameters had good discriminative accuracy, however being the most complex model, it may overfit the data as indicated by the lower calibration slope of 0.654 versus 0.824 for the expert model and 0.789 for the data driven model.

For sensitivity analysis we leveraged a separate validation cohort with an equal number of patients from each centre to exclude centre specific factors that contribute to EAO. The performance of the tested models remained comparable, with C-statistics of 0.753 and 0.722 in the data-driven and expert model respectively and 0.732 for KDRI.

Taken together, concerning the prediction of EAO, our study thus indicates that the new sparse *data-driven* and *expert-based models* tend to perform better in our cohort compared to a KDRI based model. Both the *data-driven model* with only three donor factors (age, eGFR and cause of brain death) and an *expert model* with four donor characteristics (age, eGFR, urine output and CIT) reveal moderate prediction accuracy and robust calibration that supports differentiation of favourable from unfavourable donor profiles.

## Discussion

In view of an increasing donor shortage optimal organ acceptance strategies are essential. Efforts to enhance organ utilization are currently made through multiple approaches and at a large scale. Especially in the US, the Organ Procurement Transplantation Network and the United Network for Organ Sharing have introduced several strategies. One was the implementation of the KDPI which is derived from the KDRI.

Despite its widespread use in the United States, its application is debated within the Eurotransplant region. The score may fail to capture the full complexity of the organ allocation process, leaving out important factors such as procedural details, immunological profiles, or recipient-specific characteristics. Furthermore, it has been suggested that the KDRI contributes to higher organ discard rates in the U.S., raising concerns about the effectiveness of this donor-based index (21, 22). A Canadian study by Rose et al. found that donor age alone offers predictive performance similar to the full KDRI (23).

This is in line with the present results. In this multicentric study with a cohort of 1353 transplant recipients of three different transplant centres in Germany, we investigated which donor factors can be used to predict or model the early outcome after transplantation, i.e. EAO. To this end, various models were developed and validated regarding performance and tested against each other. We performed a survey among seven senior nephrologists with years of experience within transplantation and organ allocation. Here, we found that for donor age, donor kidney function (minimal eGFR prior to donation and urine output) and for CIT there is good consensus among senior transplant nephrologists at the different centres concerning their impact on graft acceptance and early transplant outcome. Subsequent *data-driven* analysis with Lasso revealed that mainly donor age, minimal eGFR prior donation and the cause of brain death were the most relevant in predicting EAO. The *data-driven* and the *expert model* performed equally well. A third model used was integrating a variety of more donor factors (14 factors) with similar performance compared to the *expert model* but the most complexity (16 factors versus 4 (expert) and 3 (data-driven) factors. As a fourth model, the KDRI was calculated for comparison and showed a tendency for worse outcome differentiation. Likewise, European studies have highlighted the limitations of using the KDRI (24–27).

This study confirmed and refined the findings of previous studies assessing donor associated risk factors (28). The observation, that some of the findings revealed in this study have previously been reported in retrospective analysis of graft failure (for example the strong effect of donor age on early graft loss) (21), highlights the importance of a clinically guided characterisation of the graft over purely statics driven algorithms (9, 29).

This study has important limitations as this is a retrospective analysis of outcome after transplantation of donated and transplanted kidney grafts. This necessarily results in some degree of bias, as the study is restricted to the variables collected during the organ allocation process and declined or discarded grafts could not be analysed. A second limitation is the comparably high percentage of kidneys with EAO, which echoes the proportion of old donors and recipients in the German Eurotransplant population, specifically the Eurotransplant senior program (ESP). This underlines the importance of further study in a larger, but equally well characterised transplantation cohort. Also, as this is a study at German Eurotransplant centres with a mostly Caucasian and aging patient population our conclusions may not apply to patient cohorts with a different distribution of these factors. Finally, this study is potentially biased by confounders of clinical decisions at the centres and allocation criteria specific to the Eurotransplant system, such as the ESP.

One of the strengths of this study is the use of a well-curated dataset with minimal missing entries, enhancing the reliability of the analysis. This cohort closely represents the current Eurotransplant donor population, as reflected by a median donor age of 60. Additionally, the models were developed using generalised estimating equations (GEE) to account for paired data, addressing the potential correlation between grafts from the same donor transplanted into different recipients, further strengthening the robustness of the findings.

Summarising, our study demonstrates the limited benefit of the KDRI within the Eurotransplant region, where most donors fall into the highest quartile (30) and KDRI usage could lead to an unnecessarily high decline rate. Correspondingly, similar statistical models derived from multiple donor factors for prediction of early graft function are often complex. Here, we show the relevant donor factors can be reduced to a few, while maintaining similar or better accuracy in outcome prediction. Together, the clinically driven nomination of donor characteristics and the penalised regression analysis estimated donor age and the baseline donor kidney function as variables that may well be part of the donor associated substrate for EAO and could be leveraged for the development of clinical criteria for the acceptance of deceased-donor kidney grafts.

## Data Availability

The original contributions presented in the study are included in the article/**Supplementary Material**. Further inquiries can be directed to the corresponding author.
