# Supplementary material for: Evaluation of deceased-donor kidney offers: development and validation of novel data driven and expert based prediction models for early transplant outcomes
Source: Front Immunol. 2025 Jan 7;15:1511368. doi: 10.3389/fimmu.2024.1511368 (PMC11747414; doi:10.3389/fimmu.2024.1511368)
Supplement: Supplementary file 1 [file Table1.docx]

## **Supplementary Table 1**: “Full Model”

| **Variable** | **OR** | **std. error** | **p value** | **95% CI** |
| --- | --- | --- | --- | --- |
| Age | 1.029 | 0.008 | <0.001 | 1.01 to 1.04 |
| Minimal eGFR | 0.980 | 0.006 | 0.002 | 0.97 to 0.99 |
| Creatinine at explantation | 0.625 | 0.220 | 0.033 | 0.41 to 0.96 |
| Cause of brain death | 1.640 | 0.186 | 0.008 | 1.14 to 2.36 |
| Urine Output | 0.999 | <0.001 | 0.328 | 0.99 to 1.00 |
| Body-Mass-Index | 0.879 | 0.182 | 0.480 | 0.62 to 1.26 |
| Hypertension | 1.419 | 0.167 | 0.036 | 1.02 to 1.97 |
| Diabetes | 0.928 | 0.248 | 0.765 | 0.57 to 1.51 |
| Resuscitation | 0.985 | 0.006 | 0.014 | 0.97 to 0.99 |
| Smoker | 1.302 | 0.173 | 0.127 | 0.93 to 1.83 |
| Ongoing RRT | 7.448 | 0.890 | 0.024 | 1.31 to 42.44 |
| Length of Stay | 1.010 | 0.013 | 0.432 | 0.98 to 1.04 |
| Cold ischemic time | 1.004 | 0.015 | 0.778 | 0.98 to 1.03 |
| HLA mismatch | 1.103 | 0.053 | 0.062 | 0.99 to 1.22 |

*Multivariate analysis based on EAO (early adverse outcome) within 90 days post transplantation for variables selected as relevant based on the chosen model.*

**Supplementary Table 2:** Multivariate analysis “Data Driven Model“

| **Variable** | **OR** | **std. error** | **p value** | **95% CI** |
| --- | --- | --- | --- | --- |
| Age | 1.04 | 0.007 | <0.001 | 1.03 to 1.06 |
| Minimal eGFR | 0.98 | 0.004 | <0.001 | 0.98 to 0.99 |
| Cause of brain death | 1.92 | 0.181 | <0.001 | 1.34 to 2.73 |

*Multivariate analysis based on EAO (early adverse outcome) within 90 days post transplantation for variables selected as relevant based on the chosen model.*

**Supplementary Table 3:** Multivariate analysis “Expert Model”

| **Variable** | **OR** | **std. error** | **p value** | **95% CI** |
| --- | --- | --- | --- | --- |
| Age | 1.05 | 0.007 | <0.001 | 1.03 to 1.06 |
| Minimal eGFR | 0.98 | 0.004 | <0.001 | 0.97 to 0.99 |
| Urine output (ml/h) | 1.00 | <0.001 | 0.647 | 1.00 to 1.00 |
| Cold ischemia time | 1.00 | 0.014 | 0.820 | 0.97 to 1.03 |

*Multivariate analysis based on EAO (early adverse outcome) within 90 days post transplantation for variables selected as relevant based on the chosen model.*
